# New molecular tools for meningitis diagnostics in Ethiopia – a necessary step towards improving antimicrobial prescription

**DOI:** 10.1186/s12879-018-3589-4

**Published:** 2018-12-20

**Authors:** Guro K. Bårnes, Esayas Kebede Gudina, Melkamu Berhane, Alemseged Abdissa, Getnet Tesfaw, Gemeda Abebe, Siri Laura Feruglio, Dominique A. Caugant, Hannah Joan Jørgensen

**Affiliations:** 1 Division for Infection Control and Environmental Health, Norwegian Institute of Public Health, Oslo, Norway; 2 Faculty of Medicine, University of Oslo, Oslo, Norway; 3 Institute of Health, Jimma University, Jimma, Ethiopia; 4 Norwegian Veterinary Institute, Oslo, Norway; 5 Mycobacteriology Research Center, Jimma University, Jimma, Ethiopia

**Keywords:** Cerebrospinal fluid, FilmArray, Multiplex PCR, Viral meningitis, Bacterial meningitis

## Abstract

**Background:**

Meningitis remains a top cause of premature death and loss of disability-adjusted life years in low-income countries. In resource-limited settings, proper laboratory diagnostics are often scarce and knowledge about national and local epidemiology is limited. Misdiagnosis, incorrect treatment and overuse of antibiotics are potential consequences, especially for viral meningitis.

**Methods:**

A prospective study was conducted over three months in a teaching hospital in Ethiopia with limited laboratory resources. Cerebrospinal fluid (CSF) samples from patients with suspected meningitis were analysed using a multiplex PCR-based system (FilmArray, BioFire), in addition to basic routine testing with microscopy and culture. Clinical data, as well as information on treatment and outcome were collected.

**Results:**

Two hundred and eighteen patients were included; 117 (54%) neonates (0–29 days), 63 (29%) paediatrics (1 month-15 years) and 38 (17%) adults (≥16 years). Of 218 CSF samples, 21 (10%) were PCR positive; 4% in neonates, 14% in paediatrics and 18% in adults. Virus was detected in 57% of the PCR positive samples, bacteria in 33% and fungi in 10%. All CSF samples that were PCR positive for a bacterial agent had a white cell count ≥75 cells/mm^3^ and/or turbid appearance. The majority (90%) of patients received more than one antibiotic for treatment of the meningitis episode. There was no difference in the mean number of different antibiotics received or in the cumulative number of days with antibiotic treatment between patients with a microorganism detected in CSF and those without.

**Conclusions:**

A rapid molecular diagnostic system was successfully implemented in an Ethiopian setting without previous experience of molecular diagnostics. Viral meningitis was diagnosed for the first time in routine clinical practice in Ethiopia, and viral agents were the most commonly detected microorganisms in CSF. This study illustrates the potential of rapid diagnostic tests for reducing antibiotic usage in suspected meningitis cases. However, the cost of consumables for the molecular diagnostic system used in this study limits its use in low-income countries.

**Electronic supplementary material:**

The online version of this article (10.1186/s12879-018-3589-4) contains supplementary material, which is available to authorized users.

## Background

Meningitis remains a major cause of mortality and morbidity worldwide. In Ethiopia, it is an important cause of premature death and disability, being the 9th most common cause of years of life lost and loss of disability-adjusted life years [1]. In addition to the morbidity and mortality associated with meningitis, the disease also represents a huge burden on affected families and the health care system, especially in low-income countries. One case of meningococcal disease in sub-Saharan Africa has been estimated to cost the household 90 US dollar (USD), equal to 34% of the annual gross domestic product per capita, and an additional 154 USD if the disease leads to sequelae [2].

Early treatment is essential in the clinical management of meningitis. A delay in therapy negatively affects the prognosis for patients with both bacterial [3] and viral [4] meningitis/encephalitis. Empiric antimicrobial therapy is therefore initiated on suspicion, before laboratory results are available [5, 6]. Traditionally, laboratory diagnostics of meningitis have included examination of cerebrospinal fluid (CSF) for white blood cells (WBC), measurement of glucose and protein levels, and Gram staining. These are rapid, low-cost analyses but do not have satisfactory specificity alone [7, 8]. When septicemia is additionally suspected, peripheral blood WBC count is usually obtained, but this analysis also suffers from a lack of sensitivity and specificity [9, 10]. Positive bacterial cultures from CSF can provide a definite diagnosis, but may take several days and quality requirements are high. Therefore, rapid molecular diagnostics have become routine in meningitis diagnostics in high-income countries.

In many low-income countries, poor laboratory services impede the quality of health care. Ethiopia is experiencing high gross domestic product growth, but the per capita income remains one of the lowest in the world [11]. Healthcare in Ethiopia is under-resourced, and laboratory services are weak. Inadequate and delayed microbiological services foster distrust in the value of laboratory testing among clinicians, and reduce their inclination to submit clinical samples for analyses. Ultimately, this compromises patient care, prevention of infectious diseases and antibiotic stewardship.

More often than not, in Ethiopia and other low-resource settings patients receive an inadequate diagnostic workup [12] and decisions to treat meningitis are based on clinical findings alone. This approach lacks accuracy [13, 14] and ultimately results in unjustified prescriptions of antibiotics [12]. Incorrect use of antimicrobials is a recognized challenge in Ethiopia, with delayed initiation, unindicated use, duplication of broad spectrum antimicrobials and unnecessarily long duration of treatment being the major problems [15]. Fortunately, in the last few years greater attention has been paid to antimicrobial resistance also in Ethiopia, where it is becoming an increasing concern [16].

Recently, a rapid molecular diagnostic system (FilmArray, BioFire; http://www.biomerieux-usa.com/clinical/biofire-film-array) was developed for multiplex testing for the most common causes of infectious syndromes. The system is closed and integrates nucleic acid extraction and nested PCR for detection of the pathogens included in the panels. It is easy to use, has a low risk of sample contamination, requires little hands-on time and limited prior training in molecular diagnostics. For each sample, a kit that contains all necessary reagents is used, and sample preparation takes only a couple of minutes. These features are advantageous in settings with limited experience and limited facilities for molecular analyses. The main limitation for use in such settings is the high cost of test-kits.

The aim of this study was to investigate which infectious agents were responsible for meningitis in a region of Ethiopia where treatment decisions are usually made without microbiological results. We demonstrated that an easy-to-use and reliable molecular diagnostic instrument could set the ground for improving patient management and reducing usage of antimicrobials in a resource-limited setting.

## Methods

### Study design and population

This prospective study was performed between March 20th and June 20th 2017, at Jimma University Specialized Hospital (JUSH), one of the oldest public hospitals in Ethiopia, established in 1937. It is the only teaching and referral hospital in the southwestern part of the country and serves a catchment population of 15–20 million. The hospital has 600 beds and 1621 staff members, providing services for 218,095 outpatients, 16,778 inpatients, 14,207 emergencies and 5,973 deliveries yearly (data from September 2016 to September 2017). Other public health services in the catchment area include five primary hospitals, one army hospital, 118 health centres and 482 health posts.

All patients with a clinical suspicion of meningitis and from whom a CSF sample was available for analyses were eligible for inclusion. The study was run as an integrated part of routine practice and inclusion was done by the treating physicians. A written consent was given by the participants, or by parents or guardians in the case of children or unconscious patients. As samples were taken as part of routine diagnostic practice, participation in the study required no additional interventions.

Before the start of the study, a general introduction of the project aims and procedures, to the new instrument and interpretation of results was given to all medical doctors at the pediatric and internal medicine department. A four-hour training on how to perform analysis with the FilmArray was given to four dedicated JUSH staff members at the bacteriology department. Results of the PCR analysis are generated automatically by the FilmArray software and are reported as either negative, or positive with a defined species. Two senior medical doctors (study investigators) associated with the pediatric and medical wards, respectively, acted as supervisors for other physicians on the wards during the project. They were responsible for follow-up, and supported treating physicians with clinical/treatment implications of the laboratory results. The first author, a medical doctor, was present at JUSH during the project period and followed up data collection. Sample analyses, reporting of results and clinical decisions about treatment were done by local JUSH staff.

### Clinical data, treatment, routine investigations and outcome

Patient data were collected on a structured case record form. The treating physician collected the consent and clinical data at admission while microbiological analysis and results were documented by laboratory staff. Laboratory results were, according to routine at JUSH bacteriology laboratory, collected by the treating physician and the paper transcript of the laboratory result was kept in the patient chart. Results were also intended to be immediately reported to the treating physician by cellular phone. However, the latter additional reporting was not always complied with in practice. Data on treatment, routine investigations and outcome were collected by study investigators (all medical doctors), either at discharge or retrospectively within one month of discharge. Patients with none or incomplete data on treatment, routine investigations and outcome were excluded from the relevant statistical/data analyses, but included in analyses on clinical and laboratory data.

### Sample collection

CSF samples were collected in sterile glass tubes for neonates and paediatrics and in sterile plastic tubes (5 ml, Sartstedt, Germany) for adults. They were transported to the laboratory at ambient temperature within ten minutes and laboratory analyses were started within two hours, but usually within 30 min. For patients with suspected concomitant septicemia, blood was collected in blood culture bottles using BD BACTEC Peds Plus (Becton Dickinson (BD), Franklin Lakes, NJ, USA) for paediatric patients and BD BACTEC Plus Aerobic medium (BD) for adult patients.

### Conventional laboratory analyses of CSF

CSF samples were processed according to standard procedures at JUSH which include bacteriological culture, macroscopic assessment, protein and glucose measurements, manual WBC count with differential count and Gram staining. India ink staining and rapid cryptococcal antigen testing (CrAg, IMMY, OK, USA) were performed upon request. Gram stain was performed on the primary sample, usually without centrifugation due to lack of equipment and/or consumables. CSF glucose and protein measurements, were performed when reagents were available. CSF samples were cultured onto blood agar (BD) and/or chocolate agar (BD) for 72 h at 35 °C in a CO_2_-enriched incubator (candle jar). Routine bacterial identification was based on colony morphology, Gram staining and standard biochemical reactions [17].

### Molecular diagnostic of CSF

All CSF samples included in the study were analysed using the meningitis/encephalitis (ME) panel (bioMérieux, Marcy l’Etoile, France) on the FilmArray (bioMérieux,) multiplex PCR system. The closed system performs nucleic acid extraction, reverse transcription and multiplex nested PCR, automatically interprets end-point melting curve data and provides the result. The ME panel tests for genetic targets from *Escherichia coli* K1, *Haemophilus influenzae*, *Listeria monocytogenes*, *Neisseria meningitidis*, *Streptococcus agalactiae*, *Streptococcus pneumoniae*, cytomegalovirus, enterovirus, herpes simplex virus 1 (HSV-1), herpes simplex virus 2 (HSV-2), human herpesvirus 6 (HHV-6), human parechovirus, varicella zoster virus (VZV) and *Cryptococcus neoformans/gattii*. Analyses were performed according to the manufacturer’s instructions [18]. A flow-chart of the sample analysis with the FilmArray is shown in Additional file 1: Figure S1.

#### Other laboratory analysis

CSF samples from patients with suspected tuberculous meningitis (clinical suspicion, HIV-positivity, or no improvement on conventional antibiotic treatment) were tested for *Mycobacterium tuberculosis* with the GeneXpert MTB/RIF assay (Dx System Version 4.0c, Cepheid Inc., CA, US) [19]. CSF was processed as recommended by the manufacturer: a 2:1 volume of the reagent supplied with the assay was added directly to the sample. The mixture was vortexed and incubated at room temperature for 15 min. Two mL of the reagent-sample mix was then transferred to the Xpert cartridge and analysed.

Blood culture bottles were incubated in an automated blood culture incubator BACTEC FX40 system (BD) for a maximum of five days and flagged as either “negative” or “positive” for bacterial growth. Positive blood cultures were analysed using the FilmArray blood culture identification (BCID) panel (BioMerieux, France) [20], and cultured onto blood agar (BD), MacConkey agar (BD) and chocolate agar (BD), with the same routine bacterial identification as for CSF cultures.

Other investigations, such as malaria testing using peripheral blood smear and May-Grünwald-Giemsa staining, rapid testing for HIV, haematological and biochemical analyses were performed according to routine practice at JUSH.

### Data management and statistical analysis

All data were entered into a Microsoft Excel 2013 database. Statistical analyses were performed in Microsoft Excel 2013 or in GraphPad Prism 5. Two-sided tests were used and a *p*-value < 0.05 was considered statistically significant.

## Results

### Patient characteristics

**Fig. 1:**
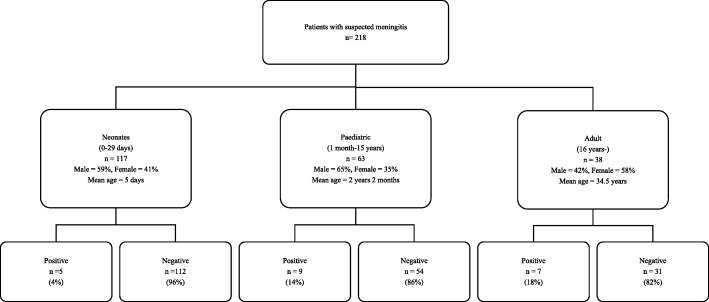
Study participants and results from analyses of cerebrospinal fluid (CSF) samples using FilmArray and culture with respect to detection of a microorganism (positive) or not (negative). Flow chart of study participants, demographics and CSF samples

During the study period, CSF samples from 220 patients with suspected meningitis were analysed at the laboratory. Of these, 218 patients gave consent and were included in the study; 42% were female. The study population consisted of 117 (54%) neonates (0–29 days), 63 (29%) paediatrics (1 month-15 years) and 38 (17%) adults (≥16 years). Demographic data and the number of samples taken per age group is shown in Fig. 1.

### Laboratory results

#### Conventional laboratory investigations

In 213 (97%) of the 218 CSF samples, the macroscopic appearance was recorded. Glucose was measured in 42 (19%) of the samples and a manual cell count was performed in 201 (92%). In 49% of these samples, no cells were detected, and 18% of the samples contained blood which prohibited the cell count. In the remaining 33% of samples WBC were detected and a differential count was available for 91% of these. Gram stain was performed in 217 samples, but microorganisms were observed in only three (bacteria in two and fungal elements in one). In total, 213 (97%) of the samples were cultured and only one sample was positive (*Klebsiella pneumoniae*) (Table 1). Hence, altogether only four (2%) of the CSF samples included in the study were found to be positive for microorganisms using conventional methods (microscopy and/or culture).

**Table 1:** Laboratory investigations of cerebrospinal fluid (CSF) samples and peripheral blood count of patients with suspected meningitis

	Microorganism detected								No microorganism detected	
	All		Virus		Bacteria		Fungi			
	n	(%)	n	(%)	n	(%)	n	(%)	n	(%)
*Macroscopic appearance of CSF*										
n	21		12		7		2		192	
Clear	11	(52)	9	(75)	1	(14)	1	(50)	133	(69)
Bloody	2	(10)	2	(17)	0	(0)	0	(0)	48	(25)
Turbid	7	(33)	0	(0)	6	(86)	1	(50)	3	(2)
Xanthochromic	1	(5)	1	(8)	0	(0)	0	(0)	8	(4)
	n	mean	n	mean	n	mean	n	mean	n	mean
*Glucose measurement (mg/mL)*	5	27	1	47	4	22	0		37	56
*Manual cell count using Neubauer chamber*	n	(%)	n	(%)	n	(%)	n	(%)	n	(%)
Total	21		12		7		2		180	
Not done due to bloody sample	1	(5)	1	(8)	0	(0)	1	(50)	35	(19)
No cells	5	(24)	5	(42)	0	(0)	0	(0)	94	(52)
Cell count ≥ 10 cells/mm3	12	(57)	4	(33)	7	(100)	1	(50)	11	(6)
Mean cells/mm3		2740		70		7716		9		91
Median cells/mm3		50		5		2500		9		0
Max. cells/mm3		40,000		332		40,000		13		9
Min. cells/mm3		0		0		75		5		0
	n	mean % PMN	n	mean % PMN	n	mean % PMN	n	mean % PMN	n	mean % PMN
*Differential count using Neubauer chamber*	14	(30)	6	(0)	7	(60)	1	(0)	45	(3)
*Gram staining/India ink staining*	n	(%)	n	(%)	n	(%)	n	(%)	n	(%)
Total	21		12		7		2		196	
No organisms seen	18	(86)	12	(100)	5	(71)	1	(50)	196	(100)
Positive	3^a^	(14)	0	(0)	2^a^	(29)	1^a^	(50)	0	(0)
*Complete blood count (CBC), peripheral blood*	n	mean	n	mean	n	mean	n	mean	n	mean
White blood count (× 10^9/L)	16	10.5	9	8.3	6	14.7	1	4.7	134	14.2
Neutrophils (× 10^9/L)	16	6.8	9	3.9	6	11.5	1	3.9	100	7.8
Lymphocyte count (× 10^9/L)	16	2.4	9	3.1	6	1.8	1	0.3	117	3.5

*PMN* polymorphonuclear leukocytes

^a^The samples positive by Gram strain had Gram-positive cocci (n = 1, CSF positive for *Streptococcus pneumoniae* on rapid molecular diagnostic (RMD), cell count 2500 (5% lymphocytes, 95% neutrophils), Gram-negative coccobacilli (n = 1, CSF positive for *Haemophilus influenzae* on RMD, cell count 40,000 with 90% lymphocytes, 10% neutrophils) and fungal elements (*n* = 1, CSF positive for *Cryptococcus neoformans/gattii* on RMD, cell count 13 with 100% lymphocytes). All three samples were negative on culture

#### Molecular diagnostics

**Fig. 2:**
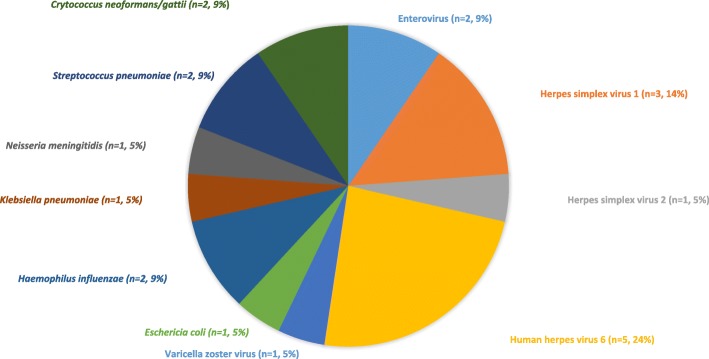
Etiological agents detected in cerebrospinal fluid (CSF) samples from patients with suspected meningitis using FilmArray and culture. Pie chart of etiological agents detected in CSF samples from patients with suspected meningitis

In 21 (10%) of the CSF samples, pathogens were detected by PCR using the FilmArray ME panel (*n* = 20) and BCID panel (*n* = 1). Using the ME panel, viral agents were detected in 57% (HHV-6, HSV-1, enterovirus, HSV-2, varicella zoster virus), bacteria in 33% (*H. influenzae, S. pneumoniae, E. coli, N. meningitidis*) and fungi in 10% (*C. neoformans/gattii*) (Fig. 2). Only one CSF sample was culture positive, but this sample was negative by FilmArray using the ME-panel. The culture was identified as *K. pneumoniae*, which is not included in the ME panel. The CSF sample was therefore analysed by FilmArray using the BCID panel, which confirmed positivity. A blood culture sample from the same patient was also *K. pneumoniae*-positive by the BCID panel.

The proportion of positive CSF samples increased with age with 4% in neonates, 14% in paediatrics and 18% in adults (Fig. 1). Table 2 shows the results of the CSF analyses and patient outcome for samples where a pathogen was detected.

**Table 2:** Age, sex, CSF analyses and outcome of patients with microorganism detected in CSF sample

			CSF analyses							
Sex	Age range	Antibiotics prior to sampling	PCR^a^	Appearance	Cell count	% Neutrophils	% Lymphocytes	Gram-stain and microscopy	Culture	Outcome
M	< 1 month	Unknown	Enterovirus	Bloody	No cells			No organism seen	Negative	Improved
F	< 1 month	No	Enterovirus	Clear	25	0	100	No organism seen	Negative	Unknown
M	1–12 months	No	Herpes simplex virus 1	Bloody	N.D^b^			No organism seen	Negative	Improved
M	> 20 years	Yes	Herpes simplex virus 1	Xanthochromic	152	0	100	No organism seen	Negative	Died
F	> 20 years	Yes	Herpes simplex virus 1	Clear	No cells			No organism seen	Negative	Unknown
F	> 20 years	No	Herpes simplex virus 2	Clear	250	0	100	No organism seen	Negative	Unknown
M	< 1 month	Yes	Human herpes virus 6	Clear	5	0	100	No organism seen	Negative	Improved
M	< 1 month	No	Human herpes virus 6	Clear	8	0	100	No organism seen	Negative	Improved
F	1–12 months	No	Human herpes virus 6	Clear	No cells			No organism seen	Negative	Improved
M	1–5 years	No	Human herpes virus 6	Clear	No cells			No organism seen	Negative	Improved
F	6–10 years	Yes	Human herpes virus 6	Clear	332	1	99	No organism seen	Negative	Improved
M	> 20 years	No	Varicella zoster virus	Clear	No cells			No organism seen	Negative	Same condition
F	> 20 years	No	*Escherichia coli*	Clear	75	60	40	No organism seen	Negative	Same condition
M	1–5 years	No	*Haemophilus influenzae*	Turbid	40,000	95	5	Gram negative rods	Negative	Improved
M	10–20 years	No	*Haemophilus influenzae*	Turbid	5200	90	10	No organism seen	Negative	Left hospital against medical advice
M	4 years	Yes	*Neisseria meningitidis*	Turbid	4200	80	20	No organism seen	Negative	Improved
M	1–12 months	No	*Streptococcus pneumoniae*	Turbid	2500	10	90	Gram positive cocci	Negative	Improved
M	10–20 years	No	*Streptococcus pneumoniae*	Turbid	1040	70	30	No organism seen	Negative	Unknown
M	< 1 month	No	Negative	Turbid	1000	10	90	No organism seen	*Klebsiella pneumoniae*	Died
F	> 20 years	No	Cryptococcus^c^	Turbid	13	0	100	Fungal elements	Negative	Died
M	> 20 years	No	Cryptococcus^c^	Clear	5	0	100	No organism seen	Negative	Unknown

*N.D*. not done

^a^ FilmArray system, meningitis-encephalitis panel, from BioMerieux

^b^ Cell count not done/not possible due to bloody samples

^c^ Sample positive on cryptococcal antigen testing

Thirty two (15%) participants had blood cultures taken. Overall 12 (38%) of these were positive including 1 of 2 samples from adults, 5 of 21 samples from paediatrics and 6 of 9 samples from neonates. Two patients were positive for microorganisms in both CSF and blood culture. One patient had *K. pneumoniae* detected in both samples, CSF by culture and blood by FilmArray, while the other had HSV-1 detected in CSF and *Enterococcus spp.* detected in blood, both by FilmArray. For positive blood cultures, routine culture confirmed bacterial isolates in all samples, although in one individual, only *K. pneumoniae* was identified by culture, while the BCID panel in addition to identifying *K. pneumoniae* also detected *Enterobacter cloacae* complex. Three positive blood cultures, in which no agent was detected by FilmArray, were positive by routine culture for *Staphylococcus s*pp, *Acinetobacter* spp. and *Salmonella* spp., respectively.

CSF samples from 20 patients, 9 paediatrics and 11 adults, were analysed for *M. tuberculosis* using the GeneXpert system, and all were negative. Three samples were tested using the cryptococcal antigen test, and two of these were positive. These two samples were also positive for *C. neoformans/gattii* by FilmArray (ME panel).

#### Other diagnostic tests

Data on testing for malaria (tested or not tested) were available for 178 patients. In total, 43 (24%) were tested, all using peripheral blood smear. Only one patient was positive, with a mixed infection of *Plasmodium falciparum* and *Plasmodium vivax*.

HIV status was known for 44 (19%) patients and 12 (27%) of these were HIV positive. Both patients with meningitis caused by *C. neoformans/gattii* had advanced stages of HIV infection.

### Laboratory results in samples where microorganisms were detected versus not detected

A comparison was made of laboratory results from analyses of CSF samples where microorganisms were detected and samples where no microorganism was detected (Table 1). There was a significant difference (OR, 28.2; 95%CI 6.4–124.6; *p* < 0.0001) for detecting a pathogen in turbid versus clear samples. In all turbid samples where a pathogen was detected, this was either bacteria or fungi. However, not all samples where bacteria or fungi were detected were turbid.

Glucose measurements were generally lower in samples with bacteria (mean 22 mg/dL) compared to samples where virus (mean 47 mg/dL) or no microorganism (mean 56 mg/dL) were detected, but the number of available measurements was low (Table 1).

For all samples where microorganisms were detected by Gram stain, a pathogen was also detected by FilmArray or culturing. All CSF samples in which bacteria were detected by PCR or culture (Table 3) had a cell count ≥75 cells/mm^3^ and/or turbid appearance. In this group of samples (≥75 cells/mm^3^ and/or turbid appearance) a bacterial agent was detected in 44%.

**Table 3:** Macroscopic appearance and leucocyte count of cerebrospinal fluid (CSF) with respect to confirmed bacterial meningitis

	CSF appearance^a^			
	Clear/Xanthochromic		Turbid	
	Total	Bacterial meningitis^b^	Total^c^	Bacterial meningitis^b^
Leukocyte count				
< 75 cells/mm3	147	0	1^d^	0
≥ 75 cells/mm3	6	1	8	6

^a^In samples containing blood, cell count was not possible. Among 50 bloody samples, none were confirmed to be bacterial meningitis

^b^Confirmed bacterial meningitis defined as bacterial agent detected on PCR or culture

^c^In one turbid sample, no cell count was performed. No microorganism was detected in this sample

^c^*C. neoformans/gattii* detected on PCR

The majority of patients had other laboratory investigations performed in addition to CSF analysis. The most common laboratory test was complete blood count in peripheral blood, but no differences were seen in mean WBC count between patients with positive and negative CSF samples (Table 1). However, patients with confirmed bacterial meningitis had a higher mean proportion of polymorphonuclear cells (PMN) in their peripheral blood.

### Clinical findings in patients where microorganisms were detected versus not detected in CSF

Table 4 shows the clinical signs and symptoms in patients with or without a microorganism detected in CSF. Fever was the most commonly reported symptom, occurring in 81% of those with a detected microorganism and 74% of those without. Headache, altered consciousness, seizure, neck stiffness, photophobia and Kernig’s and Brudzinski’s sign were more common in patients where a microorganism was detected although these characteristics could not reliably distinguish the patients. This trend was even more prominent when only patients with confirmed bacterial meningitis were considered (data not shown). Bulging fontanel was observed only in three patients, none of whom had microorganism detected in their CSF. No patient was reported to have petechiae.

**Table 4:** Signs and symptoms in patients with suspected meningitis with respect to age group and detection of microorganisms in cerebrospinal fluid (CSF)

	Microorganism detected in CSF						No microorganism detected in CSF					
	Neonates		Peadiatrics		Adults		Neonates		Peadiatrics		Adults	
	n	Mean	n	Mean	n	Mean	n	Mean	n	Mean	n	Mean
Total*	5		9		7		112		54		31	
*Vital signs*												
Temperature	5	38.2	9	38.4	6	36.9	112	37.6	54	38.3	31	37.8
Pulse	5	141	8	126	7	97	111	144	54	137	30	94
Respiratory rate	5	60	8	59	7	27	111	65	53	53	29	25
Glasgow coma scale (GSC) _/15	3	15	7	14	6	15	21	115	21	13	28	15
*Symptoms*	n	Yes (%)			No (%)		n	Yes (%)			No (%)	
Fever	21	17	(81)		4	(19)	195	145	(74)		50	(26)
Headache^a^	15	9	(60)		6	(40)	85	38	(45)		47	(55)
Nausea^a^	16	6	(38)		10	(63)	81	22	(27)		59	(73)
Vomit	21	11	(52)		10	(48)	195	59	(30)		136	(70)
Fast breathing	21	11	(52)		10	(48)	195	120	(62)		75	(38)
Shortness of breath	20	4	(20)		16	(80)	192	32	(17)		160	(83)
Cough	21	6	(29)		15	(71)	191	26	(14)		165	(86)
Stridor	21	0	(0)		21	(100)	192	3	(2)		189	(98)
Altered consciousness	21	5	(24)		16	(76)	192	31	(16)		161	(84)
Seizure	21	7	(33)		14	(67)	192	23	(12)		169	(88)
Neck stiffness^a^	16	10	(63)		6	(38)	83	26	831)		57	(69)
Photophobia^a^	16	5	(31)		11	(69)	83	11	(13)		72	(87)
Unable to feed	21	7	(33)		14	(67)	193	79	(41)		114	(59)
Abdominal pain^a^	16	1	(6)		15	(94)	84	3	(4)		81	(96)
Rash	21	0	(0)		21	(100)	191	5	(3)		186	(97)
*Signs*												
Dehydration	21	3	(14)		18	(86)	192	22	811)		170	(89)
Chest in drawing	5	2	(40)		3	(60)	110	43	(39)		67	(61)
Bulging fontanel^b^	5	0	(0)		5	(100)	110	3	(3)		107	(97)
Petecchiae	21	0	(0)		21	(100)	193	1	(1)		192	(99)
Other rash	21	2	(10)		19	(90)	192	5	(3)		187	(97)
Abdominal pain	21	1	(5)		20	(95)	188	5	(3)		183	(97)
Neck stiffness	21	8	(38)		13	(62)	190	32	(17)		158	(83)
Kernig’s sign^a^	16	6	(38)		10	(63)	82	13	(16)		69	(84)
Brudzinski’s sign^a^	16	5	(31)		11	(69)	82	8	(10)		74	(90)
Reduced consciousness	20	4	(20)		16	(8)	179	25	(14)		154	(86)

*all patients with suspected meningitis and outcome registered, also patient who had only blood culture and no CSF sample taken

^a^only applicable for paediatric and adult patients

^b^only applicable for neonatal patients

### Antibiotic treatment

Ninety-seven percent of patients received one or more antibiotic (Table 5). The most commonly used antibiotic was gentamycin, most often in combination with ampicillin, followed by ceftriaxone (data not shown). The majority (90%) received more than one antibiotic and 39% received three or more antibiotics. Broad spectrum antibiotics were used in 86% of cases (data not shown). No difference in the number of antibiotics received, duration of treatment or cumulative days of antibiotics was seen between patients with a microorganism detected and those with negative CSF (Table 5).

**Table 5:** Mean duration of antibiotic (ab) treatment and number of different antibiotics received

	Microorganism detected												No microorganism detected		
	All			Virus			Bacteria			Fungi					
	n	%	Mean duration (days)	n	%	Mean duration (days)	n	%	Mean duration (days)	n	%	Mean duration (days)	n	%	Mean duration (days)
Total^a^	16			9			6			1			153		
No. different ab															
0	0	0		0	0		0	0		0	0		5	3	
1	16	100	6	9	100	7	6	100	5	1	100	6	148	9	6
2	15	94	6	9	100	7	5	83	3	1	100	6	136	89	7
3	8	50	4	3	33	3	5	83	5	0	0		54	36	5
4	4	25	7	1	11	5	3	50	7	0	0		15	10	7
5	0	0		0	0		0	0		0	0		5	3	4
6	0	0		0	0		0	0		0	0		2	1	16
Cummulative days of ab	16		15	9		15	6		15	1		12	148		15
	n		Mean no. of antibiotics	n		Mean no. of antibiotics	n		Mean no. of antibiotics	n		Mean no. of antibiotics	n		Mean no. of antibiotics
No of antibiotics received	16		3	9		3	6		3	1		2	148		2

^a^all patients with full record of treatment and duration

### Length of stay and outcome

Data on outcome and duration of hospital stay were available for 161 (74%) and 172 (79%) of the patients, respectively (Table 6). The remaining patients were not documented in the registration book or patient files, and data could not be obtained from the ward or in the medical archive.

**Table 6:** Duration of hospital stay and outcome for patients with suspected meningitis

	Neonates						Paediatrics						Adults					
	All		Microorg. detected		No microorg. Detected		All		Microorg. detected		No microorg. Detected		All		Microorg. detected		No microorg. Detected	
*Duration of illness and length of stay*	n	Mean	n	Mean	n	Mean	n	Mean	n	Mean	n	Mean	n	Mean	n	Mean	n	Mean
Days in hospital	99	9.0	4	6.8	95	9.1	50	9.0	9	12.8	41	8.2	23	9.0	4	9.3	19	10.0
	n	%	n	%	n	%	n	%	n	%	n	%	n	%	n	%	n	%
*Outcome*	92		4		88		49		8		41		20		4		16	
Improved	66	72	3	75	63	67	39	78	7	78	32	78	10	50	0	0	10	63
Same condition	0	0	0	0	0	0	2	4	0	0	2	5	4	20	2	50	2	13
Left hospital against medical advice	12	13	0	0	12	13	3	6	1	11	2	5	2	10	0	0	2	13
Died	14	15	1	25	13	14	5	10	0	0	5	12	3	15	2	50	1	6
Patient not admitted	0	0	0	0	0	0	0	0	0	0	0	0	1	5	0	0	1	6

For patients where the duration of hospital stay was known (74%), the average length of stay was 9.0 days, and the majority (64%) of patients had improved upon discharge. There was no significant difference in length of hospital stay for patients with a causative agent identified in CSF (mean 10.0 days) versus those who had a negative CSF sample (mean 9.0 days). The overall case fatality rate was 14% and there was a trend towards a higher fatality rate in the group that had microorganisms detected in CSF (18% versus 12%), but this was not statistically significant. About 10% of the patients left the hospital against medical advice and 4% left in the same condition as when they arrived.

## Discussion

We analysed clinical and microbiological data from prospectively recruited patients with suspected meningitis in a teaching hospital in Ethiopia. The study was performed within the routine clinical and laboratory settings of a hospital that had very limited prior experience with molecular techniques. In addition to the conventional laboratory investigations used in the hospital, a simple and rapid molecular diagnostic system was introduced to enhance laboratory diagnostics during the study period. To the authors’ knowledge, this is the first time a definite etiological diagnosis of viral meningitis has been made in patients in a public health facility in Ethiopia.

Virus were the most common etiological agents of meningitis in this study with HHV-6 being the most common, followed by HSV-1 and HSV-2 and enterovirus. Although the etiology is likely to vary between age groups and local epidemiology, this is in agreement with a study from Finland that found enteroviruses, followed by HSV-2 and VZV to be major causes of aseptic meningitis in adults [21] and a study from Brazil that reported enterovirus as the most common cause of meningitis, followed by HSV-1, cytomegalovirus and dengue virus [22].

Early detection of etiologic agents improves the outcome of meningitis [23] and adequate laboratory diagnostics are imperative. Culturing of bacterial and fungal agents takes time and has low sensitivity, as illustrated by the fact that only one of 9 CSF samples with potentially cultivatable organisms was culture positive. The sensitivity of culture is affected by many factors including prior administration of antibiotics, suboptimal culturing conditions and media, and fastidious nature of some of the bacterial agents. Because of this and the unavailability of viral detection, virtually all patients with suspected meningitis in Ethiopia are treated as bacterial meningitis cases and the diagnosis is rarely re-evaluated over the course of the disease. This over-diagnosis of bacterial meningitis inevitably leads to an overuse of antibiotics. Hence, rapid molecular diagnostics can have a major impact in low-income settings by increasing the likelihood of reaching a correct diagnosis and enabling correct patient management. This is crucial not only for the outcome of the individual patient, but also for hospital biosecurity measures, public health decisions and both local and global efforts to reduce and improve antimicrobial usage.

The introduction of syndromic testing of infectious diseases and fully automated multiplexed analyses represents a paradigm shift in microbiological diagnostics [24]. Compared to the conventional laboratory investigations performed at JUSH, that detected a microorganism in CSF of only five patients (two by Gram stain, one by Gram stain and cryptococcal antigen-testing, one by cryptococcal antigen only and one by culture), the FilmArray was able to detect microorganisms in 20 samples using the ME panel. Hence, such systems can improve patient management in settings with limited laboratory facilities. The FilmArray system was very easily implemented into a modestly equipped laboratory where personnel had little prior experience with molecular diagnostics. The laboratory personnel were able to take on the extra task without disrupting their regular working schedule, while the machine operates.

However, there are a number of limitations to the sustained use of such automated systems in low-income countries. In Ethiopia, procurement of the necessary consumables is a complicated and lengthy process. The main obstacle, however, is the cost. Currently, the reagents needed for the analysis of one sample exceed 100 USD. Needless to say this is not sustainable in a public health system that is already financially constrained. On the other hand, a full course of treatment for suspected bacterial meningitis for 10–14 days [25], including only direct expenses for a hospital stay, routine investigations and antibiotic treatment, is likely to amount to more than 100 USD, even in Ethiopia. Another possible limitation of the system is the predefined selection of the pathogens in the panels. The panels were developed for an American market and may not be equally suited for Africa where other pathogens including *M. tuberculosis* and malaria are major causes of infections. It is also important that clinicians have a good understanding of test characteristics, interpretation of results and test limitations. Although lower than for conventional PCR, there is still a potential for sample contamination when using the FilmArray and the assay may detect latent or reactivated viruses [26]. The assays should be used with care and the positivity rates should be monitored.

Molecular diagnostics should not replace conventional methods. Simple investigations like WBC, Gram stain, and glucose and protein measurements provide valuable diagnostic information, helping distinguish between bacterial and non-bacterial meningitis and guiding treatment [7, 23, 27–30]. These simple methods, however, which are defined as routine investigations at JUSH, were not available for most of the study period. No patients in this study had blood/CSF glucose ratio measured, mainly because of challenges in procurement and finances leading to a lack of basic reagents. Nevertheless, the study did confirm that manual cell counts were useful and could be used more actively to influence patient management.

A combination of laboratory diagnostics and clinical examination have been shown to provide the best prediction of bacterial meningitis, with 100% sensitivity and 52% specificity [28], but even CSF cell count alone can be a good predictor. A cut-off value of 321 leukocytes/mm^3^ showed a sensitivity and specificity for bacterial meningitis of 81% in a paediatric population in Portugal [27] and all patients with bacterial meningitis in a mixed-age population in Egypt were found to have > 100 leukocytes/mm^3^ [29]. In our study, all confirmed cases of bacterial meningitis had ≥75 leukocytes/mm^3^. Thus the use of a cut-off value of ≥75 leukocytes/mm^3^ and/or a turbid appearance of CSF to indicate bacterial meningitis and the need for antibiotic treatment would have reduced the number of patients treated with antibiotics by almost 75% in this study.

The high number of patients with no cells or microorganisms detected in the CSF samples indicates that meningitis may be overestimated, especially in children and neonates. Detection of microorganisms in CSF of only 4% of neonates, might suggest that lumbar puncture, an invasive procedure, is overused. In contrast, the high proportion of positive blood cultures in this study (38%), particularly in neonates (67%) suggests that this diagnostic procedure is underused in the hospital.

A limitation of the study is that, despite close follow-up, medical files with treatment and outcome data were not traceable for a considerable proportion (27%) of the patients. This may have introduced a bias with regards to evaluating outcome. However, the proportion of patients where a microorganism was detected in CSF was the same for the overall study population (9.9%) as for the population where outcome was recorded (9.6%).

No differences were observed in this study with respect to prescription of antibiotics to patients with bacterial/fungal agents detected in CSF versus those with viral meningitis or no microorganism detected. This could be due to a lack of clinical treatment guidelines, but a contributing factor might have been poor communication of test results to responsible clinicians due to lack of electronic reporting systems, operational hospital telephones and internal post delivery systems. We acknowledge that changes in prescription habits will require long lasting interventions and coordinated stewardships in order to be successful, and that the short duration of the study was a limitation, in this respect. A recent publication from India observed a target specific escalation and evidence based de-escalation of the use of antimicrobials when a syndromic based molecular diagnostic system for meningitis was used over a period of 4 years [31].

The prescription of antibiotics is far higher in Ethiopia than WHO recommendations [32]. There is no formal antimicrobial stewardship program at JUSH, nor restrictions or specific guidelines on antimicrobials use. In Ethiopia, there is no national policy or guidelines on antimicrobials use, neither do most hospitals have their own systems to ensure correct use of antimicrobials [15]. A study from JUSH also showed that the national guidelines did not cover the management of 20% of diagnosed infectious diseases [15]. Ensuring stable access to simple CSF investigations, including protein and glucose measurements, and developing and implementing treatment guidelines for meningitis based on laboratory results might be a first and important step to improve treatment and reduce the misuse of antibiotics.

## Conclusions

In this study we diagnosed viral meningitis in a routine clinical practice for the first time in Ethiopia, and in the study population viral agents were the most common cause of meningitis. A simple, rapid molecular diagnostic system was successfully implemented in a laboratory with little experience in molecular diagnostics, and significantly increased the likelihood of detecting a microorganism in CSF samples. The lack of laboratory methods to effectively distinguish bacterial from non-bacterial causes is an important reason for the overuse of antibiotics in patients with suspected meningitis in Ethiopia. Improved diagnostics together with development of treatment guidelines, based on local epidemiology and laboratory findings, could reduce antibiotic usage and hospitalization without negatively affecting patient outcome in the study hospital.

## Additional file

Additional file 1: Flow chart of cerebrospinal fluid (CRF) sample analysis using the FilmArray system. Description of work flow for sample analysis. (DOCX 29 kb)

## Abbreviations

BCID: Blood Culture Identification
CSF: Cerebrospinal fluid
JUSH: Jimma University Specialized Hospital
ME: Meningitis/encephalitis
PMN: Plymorphonuclear leukocytes cells
USD: US Dollar
WBC: White blood cells
