# Supplementary material for: New molecular tools for meningitis diagnostics in Ethiopia – a necessary step towards improving antimicrobial prescription
Source: BMC Infect Dis. 2018 Dec 20;18:684. doi: 10.1186/s12879-018-3589-4 (PMC6302510; doi:10.1186/s12879-018-3589-4)
Supplement: Supplementary file 1 — Flow chart of cerebrospinal fluid (CRF) sample analysis using the FilmArray system. Description of work flow for sample analysis. (DOCX 29 kb) [file 12879_2018_3589_MOESM1_ESM.docx]

**Additional Figure S1**
